# Management of odonto-stomatological emergencies during the COVID-19 alarm state in dental clinics in the Autonomous Community of Madrid (CAM), Spain: An observational study

**DOI:** 10.4317/medoral.24075

**Published:** 2020-10-09

**Authors:** Juan M. Ramírez, Luís Varela-Montes, Diego Gómez-Costa, Giovanni Giovannini, Martín Romero-Maroto, Rafael Gómez de Diego

**Affiliations:** 1 Department of Morphological Sciences, School of Medicine, University of Córdoba, Spain; 2 Student of the "master de cirugía bucal e implantología" Universidad Rey Juan Carlos, Madrid, Spain; 3 Profesor of Oral Surgery at the School of Dentistry, Universidad Rey Juan Carlos, Alcorcón-Madrid, Spain; 4 Profesor at Facultad de Ciencias de la Salud, Universidad Alfonso X el Sabio, Madrid, Spain; 5 Catedrático de Ortodoncia of Oral Surgery at the School of Dentistry, Universidad Rey Juan Carlos, Alcorcón-Madrid, Spain; 6 Profesor of the “master de cirugía e implantología bucal”, Oral Surgery at the School of Dentistry, Universidad Rey Juan Carlos, Alcorcón-Madrid, Spain

## Abstract

**Background:**

Odontology practice has been severely compromised by the pandemic caused by COVID-19 and Spain is one of the countries with higher incidence. Our aim with this study is to find out the number of cases and type of odonto-stomatological emergencies (OSE) treated in four dental clinics of the Madrid capital area and region (CAM) in the period covered between March 17th and 4th of May.

**Material and Methods:**

We search the cases in the demographic/epidemiological databases of the CAM regional government and the Illustrious Official College of Dentists and Stomatologists of the First Region (Madrid).

**Results:**

We found that the most prevalent pathology was acute apical periodontitis whereas odontogenic abscess showed the lowest frequency. Prosthetic-orthodontic OSE represented 14% of cases.

**Conclusions:**

In this period of time, the most prevalent pathology acute apical periodontitis, odontogenic abscess reported the lowest frequency and prosthetic-orthodontic treatments were the third in number of cases. Most of OSE were resolved, without referring the patient to a hospital emergency department.

** Key words:**Odonto-stomatological emergencies, COVID-19, Spain.

## Introduction

On January 31, 2020, the first case of infection with the SARS-CoV-2 virus (COVID-19) was detected in Spain, from that date and until March 14, dental clinics continued with their normal activity. On March 14, the Spanish Government issues Royal Decree 463/2020 declaring the state of alarm in the national territory for the management of the health crisis caused by COVID-19, including, among other limitations, the confinement of the population in their homes, freedom of movement and suspension of non-essential activities (Bol. Of. Del Estado. 14th of March 2020:25390–25400). Previously on March 7 the Official Gazette of the C.A.M. (BOCM) adopts public health measures concerning training activities in health centres, advising its suspension because it may pose a risk to public health (Bol. Of. La Comunidad Madrid 7th of March. 2020[57]: 6–8); Likewise, the General Council of Dentists of Spain (CGDE) in a letter addressed to the Minister of Health on March 16 suggests only attending to odonto-stomatological emergencies (OSE), postponing routine dental practices (C. de Dentistas, Organ. Col. Dent. España. 2020[54]:1–2); likewise, in another letter dated March 19, to the same authority, requests that the suspension of dental activity be accompanied by the establishment of a network of clinics that prevent neglect of the population and prevent the collapse of the public health system (C. de Dentistas, Organ. Col. Dent. España. 2020[55]: 1). In response to the letter of March 16, the Secretary of the Director of the Cabinet of the Presidency of the Government, communicates that dental clinics can continue to be open if the doctors wish, establishing that they are essential services according to Order SND / 310/2020, of 31 March (Bol. Of. Del Estado. 1st of April. 2020[91]: 27984–27987). In March, the CGDE and the Madrid Regional Government of Comunidad Autónoma de Madrid (CAM) issue a series of recommendations regarding the norms and materials to be used pre and post treatment during the pandemic period (C. de Dentistas, el uso correcto de los EPIs en la atención de urgencia, Organ. Col. Dent. España. [2020] 1–2 and C. de Sanidad, Instrución técnica: correcta utilización de los equiepos de protección (EPI´s) en diferentes ubicaciones de un centro asistencial. Código:IT-EPIs CoV02-03, Comunidad Autónoma de Madrid. [2020] 1), this means that most dental consultations do not have individual protective equipment (PPE) required (1), due to market shortages, cease their clinical activity at a frequency of 95%.

Given that Spain has been severely affected by the pandemic caused by COVID-19 (2), the objective of our study is to describe the number of cases and type of OSE treatments carried out in four dental clinics of the CAM in the period covered between March 17th and 4th of May 2020, to avoid referring these patients to the CAM emergency hospital services.

## Material and Methods

We reviewed the demographic/epidemiological of medical databases of the CAM regional government and the Illustrious Official College of Dentists and Stomatologists of the First Region (Madrid). The epidemiological records associated with the pandemic in the CAM are those shown in Table 1.

The CAM has 6,778,382 inhabitants in 179 municipalities, with a population density (inhabitants/municipality) comprised between the 44 inhabitants of Robregordo and the 3,182,981 of Madrid capital (Instituto Nacional de Estadística, No Title, [2020]. https://www.madrid.org/iestadis/fijas/estructu/demograficas/padron/estructupopc_prov.htm). The dental clinics that participated in this analysis provided the numbers of OSE treatments are located in the Moncloa district (Madrid City Council) and in the municipalities of Alcorcón, Aranjuez and Navalagamella. Table 2 shows the demographic data of the described populations and the number of dental clinics.

**Table 1:**
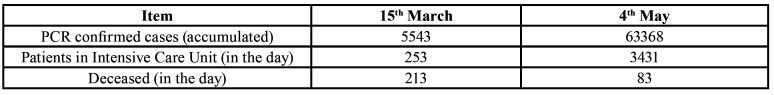
Analysis records.

**Table 2:**
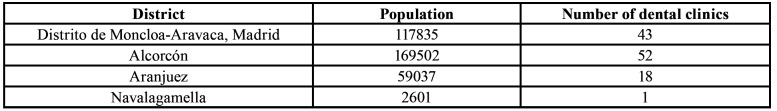
Demographic data.

The method of care was established through prior telephone appointments or via the internet, on a 24-hour schedule, 6 days a week. All the patients who requested treatment were subjected to test that were repeated when attending the consultations, in order to identify the potentially infected subjects; as well as the measurement of their body temperature, not being treated if they answered positively to any of the questions or had a temperature higher than 37º centigrade (3,4). All participants in this descriptive observational study, prior to treatment, signed an informed consent designed according to the standard one described in the Spanish law 41/2002, of November 14, that regulates the autonomy of the patient and rights and obligations regarding information and clinical documentation (5). The document was adapted following the recommendations published by the Illustrious Official College of Odontologists and Stomatologists of the 1st Region (Spain) for the care of odonto-stomatological emergencies and surgical treatment during the situation of the COVD-19 virus infection pandemic (6). Likewise, the principles established in Organic Law 3/2018, of December 5, on the Protection of Personal Data and guarantee of digital rights were followed, so that the patient could know at all times the purpose and use of that data, and who uses it. For this, the data were obtained anonymously and processed, omitting those items that could identify the subjects that make up the study sample (7).

## Results

During the referred period, 261 subjects were attended (133 women and 128 men), with ages ranging from 20 months to 87 years, 11 belonged to the child age group (0 to 14 years), 29 to the group of over 65 years, and 221 of the treated subjects belonged to the group of 15 to 65 years.

All the OSE treatments performed were protocoled according to the criteria of the 4-hand work technique, to reduce the incidence of infections and increase the efficiency of work (8); as well as with the use of high volume saliva ejectors to reduce the aerosols associated with the rotary ones (9). The professionals followed at all times the recommendations for the action described in the current scientific literature and the medical-dental societies (3,4,6). The most common treatment hours were between 9 and 24 hours, registering 25% of cases in the time slot between 24 and 6 hours.

Table 3 shows the frequency of the treated OSE, with acute and painful inflammation of the apical periodontal ligament being the most prevalent and coronary fracture without pulpal involvement having the lowest incidence. Most of the analysis subjects (75%) when attending the consultations were being treated with analgesics and/or NSAIDs (except hypertensive and diabetic patients in treatment (10).

**Table 3:**
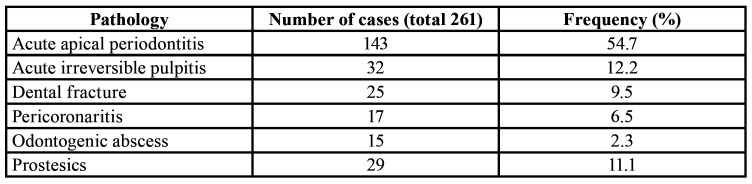
Major pathologies associated to OSE.

## Discussion

OSE are understood as the sudden appearance of a pathological bucco-maxilic conditions, which causes a spontaneous demand, the treatment of which must be immediate, urgent, timely and efficient.

It has been reported that the most common OSE is pain in the orofacial territory (87.2%), being associated with dental pathology in 52.6% of cases (11), despite the fact that acute pain of dental origin usually has some characteristics disabling for the subject who suffers from it (12), the fear of going to the emergency health centres and dental clinics due to the COVID-19 pandemic, led to an increase in the prevalence of self-medication with pain relievers and NSAIDs, this study registering a frequency of 78% compared to 68% published in the literature for the countries of Europe (13,14); the consumption of analgesics was frequently associated with the use of antibiotics, which probably minimized the painful symptoms and increased the frequency of the need for extractions (58%) as the teeth were not candidates for therapeutic restoration.

In our study periapical injury due to intraradicular infection in the acute phase registered the highest prevalence. The highest frequency of pulpitis occurred in the serous phase (20 cases), showing intense, spontaneous, continuous and irradiated pain that increased with exertion and during the night; The rest of the subjects registered pulsating pain that calmed down with the cold, diagnosing acute irreversible pulpitis in the suppurative phase. The acute infectious processes of the soft tissues surrounding the retained or impacted tooth were the most frequent in their serous form (15 cases), the rest of the treated cases -5- were suppurative pericoronaritis. All the odontogenic abscesses observed were of the periodontal type, with those associated with patients with periodontitis registering the highest frequency (93%), locating only one case of periodontal abscess due to impaction of the rest of the food (fish bone) in a subject without disease periodontal. All of the dental fractures did not have pulp involvement, being classified as uncomplicated and their pathogenesis being associated with dental trauma due to food intake (nuts).

In our cohort, 166 cases required the extraction of the affected tooth was, being necessary in 17 cases to perform scaling and root planing techniques, the rest of the subjects [95] exclusively needed a pharmacological treatment with analgesics or NSAIDs, resorting to the use of antibiotics only in 10% of the cases described.

Cases associated with prosthetic or orthodontic were 20 and 9 respectively. Among the first, the decrease in dento-supported fixed prostheses registered a frequency of 6 cases, and in particular 4 cases were associated with the loosening of the implant-retained fixed prosthesis retention prosthetic screws. The most prevalent OSE related to prosthetic cases was the fracture of the acrylic base or resin teeth of removable or implanted retained prostheses, which was observed in 10 cases. The 9 cases associated with orthodontic were related to the decrease of the retention elements (brackets), the exit of the retention arch of the support elements (bands) or presence of traumatic thrush on the cheek, tongue and lips caused by the active elements, support or retention.

## Conclusions

The use of self-medication for pain of dental origin looks like to register a higher prevalence of cases when compared with published data being the most prevalent pathology acute apical periodontitis, with odontogenic abscess being the one with the lowest frequency. Prosthetic-orthodontic OSE were the third causes of treatments. In most cases, the OSE was resolved, and it was only necessary for three patients to refer them to a hospital emergency department for possible peritonsillar, pharyngeal or diffuse phlegmon abscesses.
